# A Deep Learning Method for Quantification of Femoral Head Necrosis Based on Routine Hip MRI for Improved Surgical Decision Making

**DOI:** 10.3390/jpm13010153

**Published:** 2023-01-12

**Authors:** Adrian C. Ruckli, Andreas K. Nanavati, Malin K. Meier, Till D. Lerch, Simon D. Steppacher, Sébastian Vuilleumier, Adam Boschung, Nicolas Vuillemin, Moritz Tannast, Klaus A. Siebenrock, Nicolas Gerber, Florian Schmaranzer

**Affiliations:** 1 Personalised Medicine Research, School of Biomedical and Precision Engineering, University of Bern, 3008 Bern, Switzerland; 2 Department of Orthopaedic Surgery and Traumatology, Inselspital, University Hospital of Bern, 3010 Bern, Switzerland; 3 Department of Diagnostic-, Interventional- and Pediatric Radiology, Inselspital, University Hospital of Bern, 3010 Bern, Switzerland; 4 Department of Orthopaedic Surgery and Traumatology, Fribourg Cantonal Hospital, University of Fribourg, 1752 Fribourg, Switzerland

**Keywords:** hip, femoral head necrosis, Kerboul angle, MRI, segmentation, deep learning

## Abstract

(1) *Background*: To evaluate the performance of a deep learning model to automatically segment femoral head necrosis (FHN) based on a standard 2D MRI sequence compared to manual segmentations for 3D quantification of FHN. (2) *Methods*: Twenty-six patients (thirty hips) with avascular necrosis underwent preoperative MR arthrography including a coronal 2D PD-w sequence and a 3D T1 VIBE sequence. Manual ground truth segmentations of the necrotic and unaffected bone were then performed by an expert reader to train a self-configuring nnU-Net model. Testing of the network performance was performed using a 5-fold cross-validation and Dice coefficients were calculated. In addition, performance across the three segmentations were compared using six parameters: volume of necrosis, volume of unaffected bone, percent of necrotic bone volume, surface of necrotic bone, unaffected femoral head surface, and percent of necrotic femoral head surface area. (3) *Results*: Comparison between the manual 3D and manual 2D segmentations as well as 2D with the automatic model yielded significant, strong correlations (R_p_ > 0.9) across all six parameters of necrosis. Dice coefficients between manual- and automated 2D segmentations of necrotic- and unaffected bone were 75 ± 15% and 91 ± 5%, respectively. None of the six parameters of FHN differed between the manual and automated 2D segmentations and showed strong correlations (R_p_ > 0.9). Necrotic volume and surface area showed significant differences (all *p* < 0.05) between early and advanced ARCO grading as opposed to the modified Kerboul angle, which was comparable between both groups (*p* > 0.05). (4) *Conclusions*: Our deep learning model to automatically segment femoral necrosis based on a routine hip MRI was highly accurate. Coupled with improved quantification for volume and surface area, as opposed to 2D angles, staging and course of treatment can become better tailored to patients with varying degrees of AVN.

## 1. Introduction

Femoral head necrosis (FHN) is a significant cause of hip osteoarthritis and a disabling disease of the hip, particularly in young adults [1]. Once osteonecrosis is apparent through radiographic or clinical evidence, arthritis and collapse of the femoral head will likely occur without any subsequent intervention [2]. In fact, FHN has been shown to account for roughly 10% of all total hip arthroplasties along with 10,000 to 20,000 new cases annually in the United States alone [3]. In Asia and sub-Saharan Africa, the burden is even worse, with over 50% of total hip replacements performed being attributed to FHN [4].

Prognosis for FHN depends on the presence of a subchondral fracture of the bone, coupled with the location, size of the necrotic lesions, and stage of the disease [5]. In most cases, patients display large, necrotic lesions accompanied by femoral head fragmentation, with progression to end-stage osteoarthritis in 2–3 years. However, even smaller lesions with an intact femoral head can progress to subcortical fractures and femoral collapse, taking place in up to 50% of cases [6]. Especially problematic is the early introduction of hip prostheses in younger patients, where their higher activity levels limit the prosthetic’s durability, requiring multiple implant changes later on [7]. Alternative procedures to joint replacement for FHN include core decompression and vascularized bone grafting, which look to restore the blood supply to the femoral head [8,9]. Others include femoral osteotomies, which aim to reposition necrotic bone away from the weightbearing portion of the joint [10], and surgical hip dislocations, which provide access to the entire joint and have shown promising results for the treatment of more advanced FHN [5,11]. 

Despite these options, there is no consensus for the optimal course of action for FHN, nor in which patients with FHN will rapidly progress and whom will need surgical treatment to obviate this [6]. Currently, the revised ARCO classification, along with the Kerboul angle (used to estimate the extent of necrosis), can help prognosticate FHN [12], but these are limited to radiographs and 2D MR images. With the Kerboul angle in particular, the assessment of the size and location crucial for the grading is only semiquantitative, relying on indirect assessment and eyeballing. To date, there remains no tried and tested method to directly quantify the volume of necrosis relative to healthy bone, nor to measure the necrotic surface area in the weight bearing zone of the femoral head to incorporate into the staging [13]. This makes it very difficult to standardize surgical decision making for FHN due to the lack of rigorous evaluation and high observer-dependence [13,14].

Although staging for FHN is based on 2D imaging techniques, high-resolution 3D MRI sequences, along with the necessary graphic processing units and development of novel machine-learning based applications, should enable reconstruction of 3D MRI-based models for FHN [15]. However, to date, the feasibility of automated segmentation has not been shown yet. This would improve the spatial assessment of necrotic lesions in addition to providing a more comprehensive disease staging. Ideally, quantification of FHN from 3D models would even be based on standard 2D MRI sequences, which are universally available and performed in the routine diagnostic workup of FHN. Through integrating necrotic bone volume and surface areas to better predict which patients will benefit from reconstructive surgery for FHN, as opposed to those with too advanced necrosis, a more objective staging of FHN could be achieved. Thus, in our study, we sought to evaluate a deep-learning method to automatically quantify the necrotic bone in FHN.

Our aims were to: (1) manually reconstruct MRI-based 3D models of FHN to calculate necrotic volume and surface area to serve as a reference standard for the manual segmentations based on a 2D MRI sequence; (2) use the manual segmentations of a 2D MRI sequence for the training and testing of a neural network for automated reconstruction and quantification of FHN; and (3) compare the quantification of femoral head necrosis and Kerboul angle between early and advanced ARCO stages.

## 2. Materials and Methods

This was an IRB-approved retrospective study of 26 patients (mean age 30 years, 14 men) with FHN diagnosed in a tertiary orthopedic university hospital. Diagnosis of FHN was established in patients with a history of hip symptoms at clinical examination. All patients underwent biplanar radiographic imaging with supine AP pelvis views and cross table lateral view and subsequent MRI of the hip. FHN was graded according to the commonly recommended 2019 ARCO grading [12]: I (negative x-rays): two hips; II (no fracture): four hips; IIIA (head collapse < 2 mm): 13 hips; IIIB (head collapse > 2 mm): 11 hips. Patients underwent preoperative MR arthrography at 3T (Skyra, Siemens Healthineers, Erlangen, Germany) for their hips including the application of traction according to a previously described technique [16,17]. This included the acquisition of multiplanar proton-density (PD) weighted turbo spin-echo (TSE) imaging without fat saturation (coronal, radial and axial orientation) and a high-resolution axial-oblique 3D T1-weighted volume interpolated breath-hold examination (VIBE) sequence [18]. Sequence parameters for the coronal PD-w sequence were repetition time (TR)/echo time (TE), 2600/11 milliseconds (ms), slice thickness of 2 millimeters (mm), 170 × 170 mm field of view, matrix size of 269  ×  384, acquisition time (AT) of 3 min. Sequence parameters for the 3D T1-w VIBE sequence were TR/TE, 15/3.3 ms, slice thickness of 0.8 mm, 160 × 160 mm field of view, matrix size of 192 × 192, and an acquisition time of 8:46 min. 

Modified Kerboul angles were measured for each of the patients from the MR images, according to the method of Ha et al., where the greatest extension was assessed in the midcoronal and midsagittal planes and summed, since measuring from only the coronal plane is not as accurate in the quantification of necrosis [19]. Additionally, Tönnis scores to assess the degree of hip osteoarthritis were included, with grades from 0 (no osteoarthritis present) to 3 (large cysts, avascular necrosis, and severe narrowing of joint space) [20,21] (Table 1).

### 2.1. Manual and Automatic Segmentation of FHN

**Figure 1:**
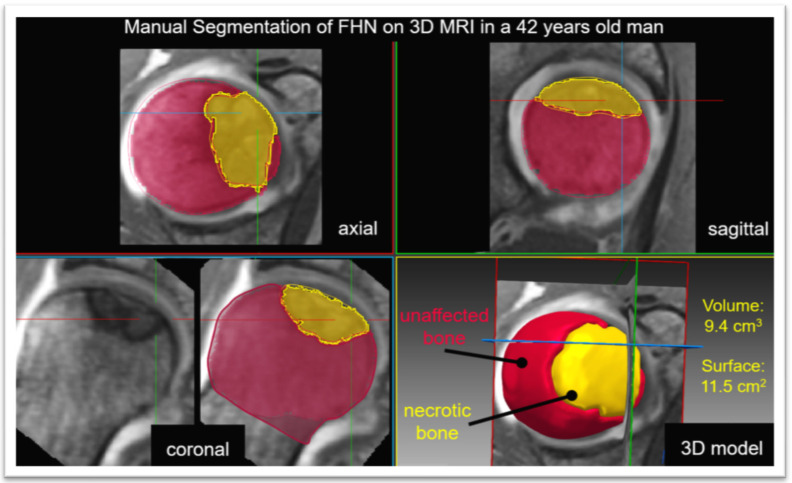
Manual segmentation of femoral head necrosis based on the 3D T1 volume interpolated breath-hold examination (VIBE) sequence is shown. The 3D sequence allows for multiplanar reformation for the threshold assisted 3D segmentation of the unaffected bone (red) and the necrotic bone (yellow). In this patient, this yielded a necrotic volume of 9.4 cm^3^ and surface area of 11.5 cm^2^.

**Figure 2:**
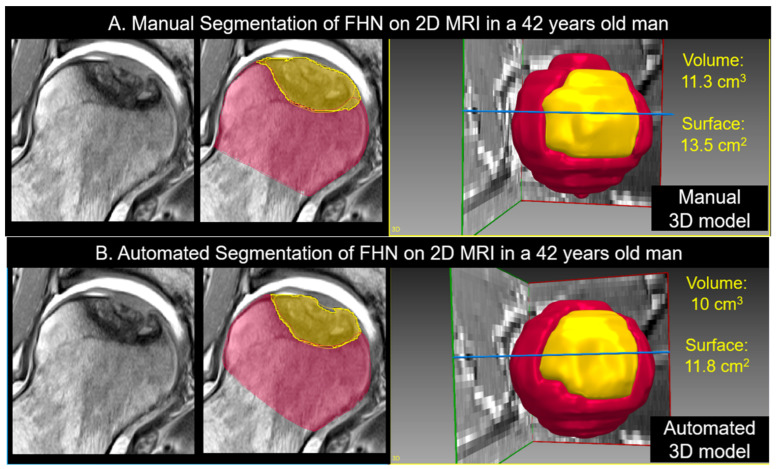
(**A**) Manual and (**B**) automatic segmentation of femoral head necrosis based on the 2D PD-w TSE sequence of the same patient as in Figure 1 is shown. Unaffected (red) and necrotic bone (yellow) were masked using threshold assisted (**A**) manual segmentation, which was used as the ground truth to train the neuronal network for (**B**) fully automatic segmentation. Automatic segmentation yielded comparable values as manual segmentation for relative necrotic volume (10 cm^3^ vs. 11.3 cm^3^) and relative necrotic surface area (11.8 cm^2^ vs. 13.5 cm^2^). Dice coefficient for necrotic bone was 90% and 94% for the unaffected bone.

Manual segmentation of the necrotic bone and unaffected femoral head was performed by an expert reader on 3D T1 VIBE MRI and the coronal 2D PD-w sequence using Amira software (FEI; Hillsboro, Oregon, USA). The manual segmentations were then used to train a set of convolutional neural networks (nnU-Net) [22] (Figure 1 and Figure 2). The neuronal network was tested with a 5-fold cross-validation scheme on the unseen data. The 5-fold cross-validation trains five different networks where 4/5 of the data are used to train and the remaining 1/5 to test the network. This has the advantage that the overall set can be used as unseen data in this configuration. Therefore, the ensemble of the five different networks built in the nnU-Net framework was not used. The architecture tested consisted of an ensembled 2D-3D U-Net that was applied on the coronal 2D PD-w TSE sequence. For the supervised deep learning approach, the manually segmented images were used as the ground truth, and the mean Dice coefficient was calculated.

Images were volume-cropped with a spacing of 160 × 30 × 160 voxels and 0.44 × 2.4 × 0.44 mm.

The network was trained for 60 epochs. Otherwise, the default settings were kept.

The volume and surface of the necrotic and unaffected region were calculated for the manual and the automatic segmentations from the neural network. The percent of necrotic bone volume and necrotic femoral head surface were calculated. 

To calculate the surface, the segmentation was converted into a contour, and a plane was fitted to the flat portion where the segmentation ends in the femoral neck. Everything within a distance of 3 mm to the plane was removed and was not part of the surface of the femoral head. Then, the surface was calculated for the overall femoral head and the necrotic part.

### 2.2. Statistical Analysis

Dice coefficients were calculated to assess the accuracy of the automatic segmentation. The mean difference between the two manual segmentations plus the difference between the 2D manual segmentation and the automatic ones were compared with the paired *t*-tests and the correlation was assessed with the Pearson correlation coefficients. We then compared the absolute and relative size of the necrosis between early and advanced stages of AVN (ARCO I/II versus IIIA/B) using Mann–Whitney U tests. A *p*-value less than 0.05 determined the statistical significance. Pearson correlations were also run for the six parameters for each segmentation relative to the modified Kerboul angle.

## 3. Results

### 3.1. Manual Segmentation of 3D MRI versus Manual Segmentation of 2D MRI

Upon direct comparison, the ground truth manual segmentation of 3D MRI was consistent with the manual segmentation of 2D MRI. The mean differences and 95% confidence intervals (CI) between the 3D and 2D segmentations were 0.08 ± 2.8 cm^3^ and −0.97 to 1.1 cm^3^ (volume of necrosis), 1 ± 6% and −2 to 3% (percent of necrotic bone volume), 0.4 ± 3.3 cm^2^ and −0.9 to 1.6 cm^2^ (surface of necrotic bone), −0.5 ± 4.2 cm^2^ and −2.1 to 1.1 cm^2^ (unaffected femoral surface), and 1 ± 5% and 1 to 3% (percent of necrotic femoral head surface). Each of these five parameters had *p*-values above the 0.05 threshold, except for the sixth parameter (volume of unaffected bone), which had a mean difference (*p* = 0.0234) and CI of –1.5 ± 3.4 cm^3^ and –2.7 to −0.2 cm^3^, respectively. Furthermore, the correlations between the 3D and 2D segmentations for all six parameters were strong (R_p_ > 0.9), with *p* < 0.001 (Table 2 and Table 3).

### 3.2. Manual Segmentation of 2D MRI Versus Automatic Segmentation of 2D MRI

Accuracy of the automatic segmentation as assessed with Dice coefficients for the automatic model were 75 ± 15% and 91 ± 5% for the necrotic and unaffected bone, respectively. Upon direct comparison, the manual segmentation of 2D MRI was comparable with the automatic segmentation of 2D MRI (all *p* > 0.05). The mean differences and confidence intervals (CI) between the segmentations were 0.9 ± 2.7 cm^3^ and –0.1 to 1.9 cm^3^ (volume of necrosis), −0.8 ± 2.8 cm^3^ and −1.8 to 0.3 cm^3^ (volume of unaffected bone), 2 ± 5% and 0 to 4% (percent of necrotic bone volume), 1.5 ± 4 cm^2^ and −0.01 to 3 cm^2^ (surface of necrotic bone), 0.7 ± 2.6 cm^2^ and −0.3 to 1.6 cm^2^ (unaffected femoral surface), and 3 ± 7% and 0 to 5% (percent of necrotic femoral head surface). Each of these six parameters showed strong correlations between the segmentations (R_p_ > 0.9), with *p* < 0.001 (Table 2 and Table 4).

### 3.3. Quantitative Comparison of Early and Advanced Stages of Femoral Head Necrosis

No significant difference (*p* = 0.0775) was observed for the modified Kerboul angle between hips with early versus advanced FHN (median of 153°, interquartile range of 58° versus 195°, 70°) (Table 5).

For the manual 2D segmentation, examination of the six aforementioned parameters between early (ARCO 0-II) and advanced (ARCO > II) FHN demonstrated significant differences (all *p* < 0.05) for volume of necrosis, percent of necrotic bone volume, necrotic bone surface, and percent of necrotic femoral head surface. Accordingly, the median and (interquartile ranges) reported for these parameters between early and advanced stages were 2.2 (2.7) vs. 8.9 (10.1), 4 (8) vs. 15 (16), 4.5 (4) vs. 12 (13.6), and 8 (10) vs. 20 (26) (Table 5).

Automatic 2D segmentation analysis followed the pattern of the manual 2D segmentation, in which significant differences (*p* < 0.05) were found for the volume of necrosis, percent of necrotic bone volume, necrotic bone surface, and percent of necrotic femoral head surface between early and advanced stages of FHN. The median and (interquartile ranges) reported for these parameters between the early and advanced stages were 2.2 (4) vs. 8.8 (7.9), 6 (6) vs. 13 (16), 4.8 (3.6) vs. 12 (8.2), and 9 (4) vs. 21 (12) (Table 5).

**Figure 3:**
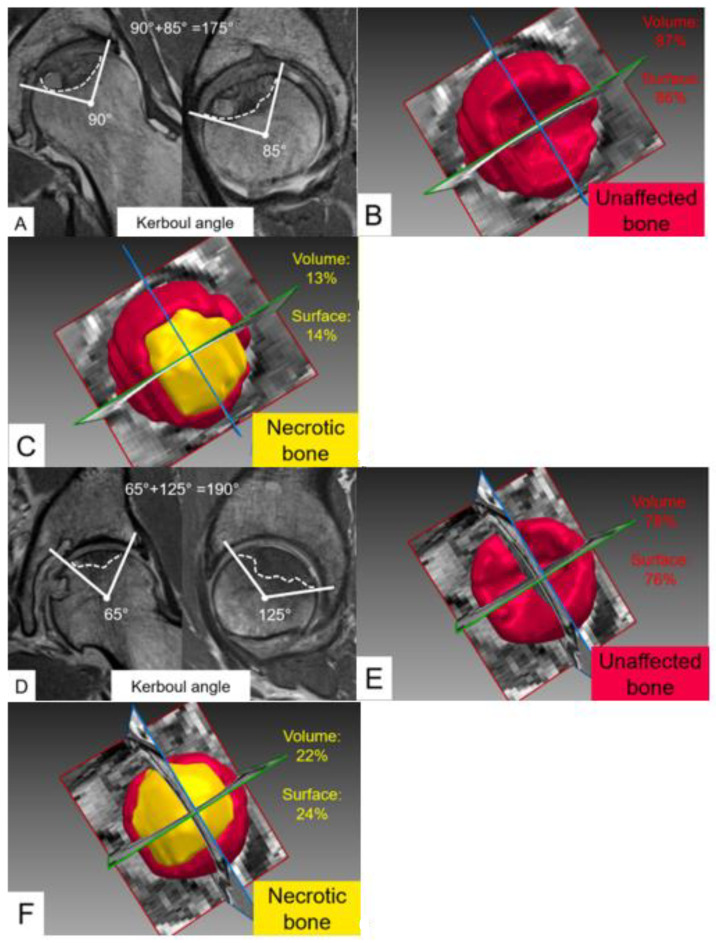
Quantification of femoral head necrosis with modified Kerboul angles and 3D quantification using fully automated 3D models of the femoral head in two different patients. (**A**–**C**) 43-year-old man and (**C**–**F**) 36-year-old woman with advanced femoral head necrosis (ARCO 3A) and comparable Kerboul angles of (**A**) 175° and (**D**) 190°. In contrast, marked differences were observed between both patients for (**C**,**F**) relative necrotic volume (13% versus 22%) and relative necrotic surface area (14% versus 24%), underlining the potential of deep learning-based 3D quantification to improve surgical planning.

Additional correlation with the modified Kerboul angle was assessed for each of the three segmentations (manual 3D, manual 2D, and automatic 2D) across the four parameters of necrosis quantification. Strong correlations were present (R_p_ > 0.85) for the volume of necrosis, percent of necrotic bone, surface of necrotic bone, and percent of necrotic femoral head surface, with all correlations being significant (*p* < 0.001) (Table 6). Despite these high correlations between modified Kerboul angles and 3D quantification of FHN, we observed marked differences in the relative necrotic volume and relative necrotic surface area in some patients with comparable Kerboul angles (Figure 3).

## 4. Discussion

In its progression, FHN leads to the collapse of the femoral head in a large number of patients, with 67% developing collapse even without the manifestation of clinical symptoms [12,23]. Accurate disease staging is thus imperative for FHN to dictate the right course of treatment, particularly in younger patients who may be able to avoid total hip arthroplasty and preserve the native joint [24]. Within our work, we sought to expand upon the current FHN staging and Kerboul angle through the inclusion of 3D volumetric and surface area quantification of necrotic lesions. We retrospectively analyzed the MRIs of 26 patients (30 hips) with FHN and varying ARCO stages upon which we performed the manual segmentation of FHN based on a 3D T1-w sequence. The segmentation of the 3D sequence served as the reference standard as it has high-spatial resolution with thin and continuous slices alike. Since numerous different 3D MRI sequences are available and not routinely performed in the workup for FHN, we further compared segmentation accuracy using the standard 2D PD-w TSE sequence and subsequent automatic segmentation using a supervised deep learning approach. Indeed, we could show that accurate quantification of FHN was possible when performed manually and fully automatically on 2D MRI **(**Table 2, Table 3 and Table 4). To the best of our knowledge, our study is the first to show that deep learning based segmentation is accurate for the 3D quantification of femoral head necrosis. Previous studies were successful in being comparable to orthopedic surgeons in diagnosing necrosis [25,26,27], with one study in particular utilizing 3D MRIs to allow for a potentially earlier diagnosis of femoral necrosis [25]. Our study also demonstrated this, as we found it to be equivalent across the six parameters of necrosis (Table 4) and correlates equally well with the modified Kerboul angle as the other manual segmentations (Table 6). This shows that not only was the automatic model accurate, but volumetric parameters (such as volume of necrosis and percent of necrotic bone) and surface ones (surface of necrotic bone and percent of necrotic femoral head surface) could be just as viable to predict necrosis as the modified Kerboul angle parameter.

Kerboul angle, as first described by Kerboul et al. [28], is a method to evaluate the total necrotic angle from lateral and anterior-posterior radiographs, which was then improved upon with the advent of the MRI [29]. The modified Kerboul angle is now typically used to assess the extent of necrosis to predict future collapse, being the sum of the necrotic angles in the coronal and sagittal planes on the MRI, and has shown promise [30]. However, as Steinberg et al. pointed out in their study, the modified Kerboul angle is more variable than parameters such as the index of necrosis and the modified index of necrosis, even when assuming a percentage of femoral head involvement from a 250-degree angle for the head rather than the 180-degree angle, which was previously implemented [31,32]. Our results seem to support the notion that the modified Kerboul angle is a less sensitive metric relative to the 3D volumetric assessment, given that the volume of necrosis, percent of necrotic bone volume, necrotic bone surface, and percent of necrotic femoral head were all significantly different between early and advanced staged ARCO whereas the Kerboul angle was not (Table 5). Although our sample size was not sufficient to perform subgroup analysis between hips with focal or more extensive FHN, we could observe marked differences when performing automated quantification of FHN compared to measuring the Kerboul angles alone (Figure 3). Other studies have also demonstrated the difficulty in measuring the actual size of a 3D lesion with 2D angular measurements [31,33,34]. 

Currently, the ARCO classification is widely utilized to distinguish between the different stages of AVN based on MRI, radiograph, and the degree of femoral depression in the more advanced stages [12]. However, 3D volumetric assessment was not incorporated in this analysis to better categorize the stages, which could prove invaluable in ensuring that patients undergo the essential surgical course [35]. Based on our results, the quantification for volume and surface area were more sensitive than the Kerboul angle, and should be used to make ARCO staging and AVN diagnosis more robust clinically. Furthermore, our results confirm that the amount of necrosis in terms of volume and surface area increased from the threshold of ARCO stage II, indicating that once collapse takes place, there is indeed a substantial change in the joint (Table 5) [36]. 

Our study had some limitations, the most significant being that all of the patient scans were acquired with the same site and same MRI vendor. As has been pointed out in previous deep learning studies, sufficient training of these models requires scans from multiple MR machines and sites to improve generalizability [37,38]. Our study paves the way for future work implementing this approach at multiple sites with scanners from different vendors, as we have demonstrated that our automatic segmentation could perform equally well to our ground truth segmentations in the quantification of FHN. Another limitation is our sample size, which although somewhat smaller due to the lower prevalence of disease, should be expanded in follow-up studies to both improve the performance of the model and ensure there is no overfitting or undertraining [36]. Finally, our study was retrospective in nature, leading to potential selection bias with the number of hips included that were in the advanced ARCO stage (IIIA or IIIB) relative to the early stages (ARCO I and II). This could have skewed some of the values obtained for the six parameters for the manual segmentations and the automatic model. 

In conclusion, our deep learning model for AVN proved to be just as accurate as our ground truth and is the first to accurately quantify necrosis based on 3D models of the femur. Such models could be further used for 3D printing or finite element analysis to better simulate the effect of different surgical approaches for treatment of the necrotic lesion. Furthermore, we were able to corroborate the findings of previous studies that the modified Kerboul angle is not the most sensitive metric, and proposed/identified four parameters that outperformed it when distinguishing between early and advanced necrosis. While larger and more heterogeneous studies need to be carried out as well as continued improvement of AVN staging, this study will hopefully allow for further work to optimize surgical decision making and ameliorate patient outcomes with the disease in the near future.

## Figures and Tables

**Table 1:** Demography and radiography of the study population.

Parameter	Mean ± SD/Number of Hips (%)
Patients (hips)	26 (30 hips)
Age (Mean ± SD)	30 ± 7
Sex (male in%)	53.85
Etiology, hips	
Idiopathic (%) Posttraumatic (%) Systemic (%) Perthes Disease (%) Treatment, hips (%) Non-Operative Treatment Hip Arthroscopy Surgical Hip Dislocation (total) Concomitant Femoral Osteotomy Periacetabular Osteotomy First Surgery: Total Arthroplasty	13 (43) 3 (10) 11 (37) 3 (10) 12 (40) 0 (0) 14 (47) 5 (16) 1 * (3) 3 (10)
Tönnis grade of osteoarthritis, hips (%)	
Tönnis grade < 2	28 (93)
Tönnis grade ≥ 2	2 (7)
ARCO grading	
ARCO I (%) ARCO II (%) ARCO IIIA (%) ARCO IIIB (%)	2 (7) 4 (13) 13 (43) 11 (37)
Modified Kerboul angle (Mean ± SD°)	198 ± 77

Values are expressed as the Mean ± Standard Deviation or as the number of hips and the percentage of the total; ARCO = Association Research Circulation Osseous Staging for osteonecrosis of the femoral head; * = surgical hip dislocation was also performed for this hip.

**Table 2:** Quantification of femoral head necrosis based on manual segmentations of 3D and 2D MRI and the automatic segmentation of 2D MRI using deep learning.

Parameter	Manual Segmentation of 3D MRI		Manual Segmentation of 2D MRI		Automatic Segmentation of 2D MRI	
	Mean ± SD	Range	Mean ± SD	Range	Mean ± SD	Range
Volume of necrosis (cm^3^)	8.9 ± 7.4	0.7 to 29	8.8 ± 7.4	0.6 to 28	7.9 ± 6.3	0.9 to 23
Volume of unaffected bone (cm^3^)	39 ± 15	20 to 72	41 ± 15	20 to 71	42 ± 14	22 to 73
Percent of necrotic bone volume (%)	19 ± 15	2 to 59	18 ± 15	1 to 54	16 ± 13	2 to 47
Surface of necrotic bone (cm^2^)	14 ± 9.3	1.3 to 35	13 ± 9.5	1.6 to 38	12 ± 8.2	0.8 to 33
Unaffected femoral head surface (cm^2^)	59 ± 14	36 to 90	59 ± 13	42 to 87	59 ± 12	44 to 84
Percent of necrotic femoral head surface (%)	23 ± 15	2 to 58	23 ± 16	3 to 60	20 ± 14	2 to 54

Values are expressed as Mean ± Standard Deviation; cm = centimeters.

**Table 3:** Comparison of the manual segmentation of femoral head necrosis on 3D MRI with the manual segmentation of 2D MRI.

Parameter	Difference (Mean ± SD)	CI	*p* Value	Correlation	*p* Value
Volume of necrosis (cm^3^)	0.08 ± 2.8	−0.97 to 1.1	0.873	R_p_ = 0.928	<0.001
Volume of unaffected bone (cm^3^)	−1.5 ± 3.4	−2.7 to −0.2	0.0234	R_p_ = 0.975	<0.001
Percent of necrotic bone volume (%)	1 ± 6	−2 to 3	0.526	R_p_ = 0.928	<0.001
Surface of necrotic bone (cm^2^)	0.4 ± 3.3	−0.9 to 1.6	0.536	R_p_ = 0.938	<0.001
Unaffected femoral head surface (cm^2^)	−0.5 ± 4.2	−2.1 to 1.1	0.515	R_p_ = 0.958	<0.001
Percent of necrotic femoral head surface (%)	1 ± 5	1 to 3	0.467	R_p_ = 0.940	<0.001

Difference values are expressed as Mean ± Standard Deviation; CI are the 95% confidence intervals; R_p_ denotes the Pearson correlation coefficient.

**Table 4:** Comparison of manual versus automatic segmentation of FHN based on 2D MRI.

Parameter	Difference, Mean ± SD	CI	*p* Value	Correlation	*p* Value
Volume of necrosis (cm^3^)	0.9 ± 2.7	−0.1 to 1.9	0.0858	R_p_ = 0.936	<0.001
Volume of unaffected bone (cm^3^)	−0.8 ± 2.8	−1.8 to 0.3	0.152	R_p_ = 0.982	<0.001
Percent of necrotic bone volume (%)	2 ± 5	0 to 4	0.10	R_p_ = 0.935	<0.001
Necrotic bone surface (cm^2^)	1.5 ± 4	−0.01 to 3	0.0517	R_p_ = 0.910	<0.001
Unaffected femoral head surface (cm^2^)	0.7 ± 2.6	−0.3 to 1.6	0.173	R_p_ = 0.979	<0.001
Percent of necrotic femoral head surface (%)	3 ± 7	0 to 5	0.0641	R_p_ = 0.892	<0.001

Difference values are expressed as Mean ± Standard Deviation; CI are the 95% confidence intervals; R_p_ denotes the Pearson correlation coefficient.

**Table 5:** Comparison of the manual and automatic segmentation of femoral head necrosis based on 2D MRI between hips with early versus advanced disease stages.

Parameters	Manual 2D Segmentation Median (IQR)			Automatic 2D Segmentation Median (IQR)		
	ARCO 0-II	ARCO > II	*p*-Value	ARCO 0-II	ARCO > II	*p*-Value
Modified Kerboul angle (°)	153 (58)	195 (70)	0.0775	153 (58)	195 (70)	0.0775
Volume of necrosis (cm^3^)	2.2 (2.7)	8.9 (10.1)	0.0133	2.2 (4)	8.8 (7.9)	0.0257
Volume of unaffected bone (cm^3^)	40 (12.2)	36 (19.2)	0.315	41 (9.9)	37 (12.5)	0.270
Percent of necrotic bone volume (%)	4 (8)	15 (16)	0.0226	6 (6)	13 (16)	0.0199
Necrotic bone surface (cm^2^)	4.5 (4)	12 (13.6)	0.0152	4.8 (3.6)	12 (8.2)	0.0133
Unaffected femoral head surface (cm^2^)	54 (12)	55 (16)	0.713	52 (12)	55 (16)	0.825
Percent of necrotic femoral head surface (%)	8 (10)	20 (26)	0.0257	9(4)	21 (12)	0.0116

IQR = interquartile range; ARCO (Association Research Circulation Osseous Staging) where >2 indicates an advanced stage of femoral necrosis.

**Table 6:** Correlations between the manual segmentation of 3D and 2D MRI and automatic segmentation of 2D MRI against the modified Kerboul angle.

Parameter	Manual Segmentation of 3D MRI		Manual Segmentation of 2D MRI		Automatic Segmentation of 2D MRI	
	vs Modified Kerboul	*p*-Value	vs Modified Kerboul	*p*-Value	vs Modified Kerboul	*p*-Value
Volume of necrosis (cm^3^)	R_p_ = 0.859	<0.001	R_p_ = 0.865	<0.001	R_p_ = 0.867	<0.001
Percent of necrotic bone volume (%)	R_p_ = 0.883	<0.001	R_p_ = 0.896	<0.001	R_p_ = 0.913	<0.001
Surface of necrotic bone (cm^2^)	R_p_ = 0.861	<0.001	R_p_ = 0.869	<0.001	R_p_ = 0.866	<0.001
Percent of necrotic femoral head surface (%)	R_p_ = 0.881	<0.001	Rp = 0.881	<0.001	R_p_ = 0.909	<0.001

R_p_ denotes the Pearson correlation coefficient.

## Data Availability

As per the national guidelines, sharing of data was not applicable.
